# Ruptured Jejunal Diverticulum Due to a Single-Band Small Bowel Obstruction

**DOI:** 10.1100/tsw.2008.118

**Published:** 2008-09-30

**Authors:** Rajaraman Durai, Ashish Sinha, Mihir Khan, Happy Hoque, Rajab Kerwat

**Affiliations:** ^1^ Department of Surgery, Queen Mary's Sidcup NHS Trust, Sidcup, Kent, UK; ^2^ Department of Pathology, Queen Mary's Sidcup NHS Trust, Sidcup, Kent, UK

**Keywords:** jejunal diverticulum, stricture, peritonitis, interloop abscess

## Abstract

Jejunal diverticulosis is rare and often goes unnoticed until complications occur. The diverticula are true, acquired diverticula and often asymptomatic. Jejunal diverticulosis can be associated with diverticulosis of the duodenum, ileum, and colon. Here we describe a patient with known severe diverticular disease of the large bowel, who presented acutely with abdominal pain and signs of generalised peritonitis. Laparotomy showed ruptured jejunal diverticulosis with a single band over the terminal ileum, causing small bowel obstruction. Spontaneous perforation of a jejunal diverticulum is rare and is usually an intraoperative finding. One should exclude a precipitating cause, such as coexisting distal obstruction, stricture, or a foreign body.

## INTRODUCTION

Jejunal diverticulosis is rare and often goes unnoticed until it causes complications[1,2]. It usually manifests in the elderly[3,4]. The diverticula are a form of true diverticula containing all layers of the small bowel. In contrast to Meckel's diverticulum, which is a true diverticulum presenting at the antimesenteric border, jejunal diverticula present along the mesenteric border. Ten percent of patients with small bowel diverticulosis may require surgical intervention for complications[5]. Here we describe a patient with known severe diverticular disease of the large bowel, who presented acutely with generalised peritonitis and signs of systemic sepsis. Laparotomy showed ruptured jejunal diverticulosis secondary to a distal obstruction.

## REPORT OF A CASE

An 84-year-old gentleman presented to the accident and emergency department with a 1-week history of increasing lower abdominal pain. He denied any change in bowel or bladder habits, or other gastrointestinal symptoms. He previously had a barium enema (Fig. 1), which showed severe diverticular disease of the sigmoid colon and his past medical history included coronary artery bypass grafting for ischemic heart disease. He was on aspirin and bendrofluazide. He did not smoke or drink. On examination, he looked

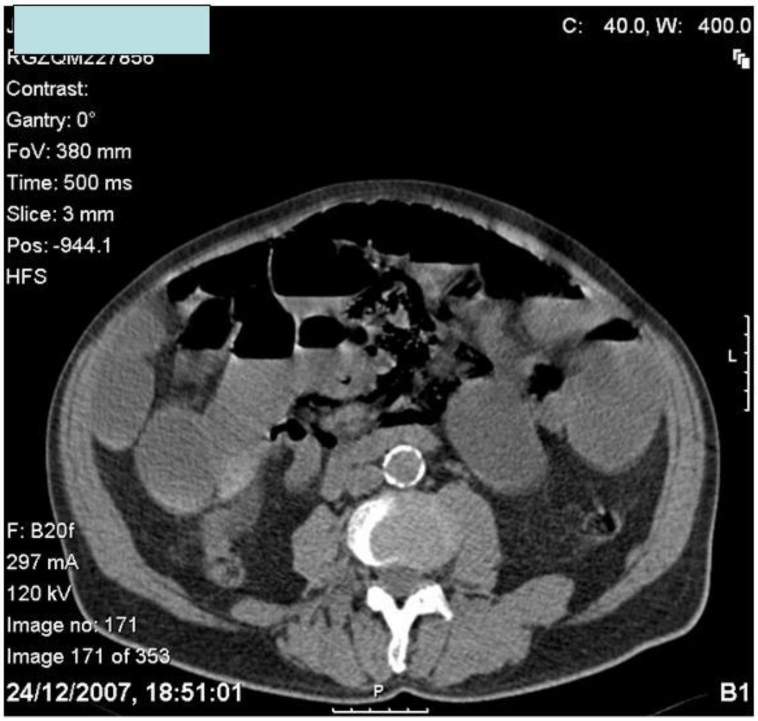

**FIGURE 1.** Barium enema showing severe diverticular disease of the sigmoid colon.

unwell, had a temperature of 36.7ºC, pulse rate of 110 beats/min, and blood pressure of 110/70 mmHg. He was clinically dehydrated, his chest was clear, and the abdomen was slightly uniformly distended. Although it was soft, he was tender in both iliac fossae, with guarding and tinkling bowel sounds. His blood tests showed a raised white cell count of $18 \times 10^9$/l with a neutrophilia, elevated CRP of 400 mg/l, urea of 44 mmols/l, and creatinine of 300 mmols/l. His remaining blood tests were within the normal range. Abdominal X-ray showed dilated small and large bowel loops (Fig. 2). His chest X-ray was suggestive of possible free air under the right hemidiaphragm (Fig. 3). A CT scan of the abdomen without contrast confirmed pneumoperitoneum and demonstrated proximal small bowel dilatation (Figs. 4 and 5).

He underwent an emergency laparotomy which revealed ruptured jejunal diverticulosis (Fig. 6) causing inter–small bowel loop abscess. The proximal half of the small bowel was dilated, congested, and oedematous. There was a band across the distal small bowel, causing small bowel obstruction, which led to rupture of the proximal jejunal diverticulum. At the site of the band, there was a stricture. The section of small bowel that contained multiple jejunal diverticula was resected and a jejuno-jejunal anatomosis was performed with a GIA stapler 75. The stapled ends were reinforced with interrupted vicryl seromuscular stitches. The stricture was also resected and a side-to-side stapled anastomosis was performed. After thorough peritoneal washout, the abdomen was closed by a mass closure with loop PDS and skin clips.

## DISCUSSION

The incidence of small bowel diverticulosis is 0.3–2.5%[6,7], and is commonly found in the 6$^{th}$ or 7$^{th}$ decade[8]. The diverticula are true (Fig. 7), acquired diverticula and often asymptomatic. Jejunal diverticulosis can be associated with diverticulosis of the duodenum, ileum, and colon[9,10]. Jejunal diverticulosis can also be associated with jejunal lipomatosis. In our case, histological examination of the resected specimen showed multiple jejunal diverticula. One of the diverticula perforated and showed all layers of the intestine. Another intact diverticulum contained undigested food particles.

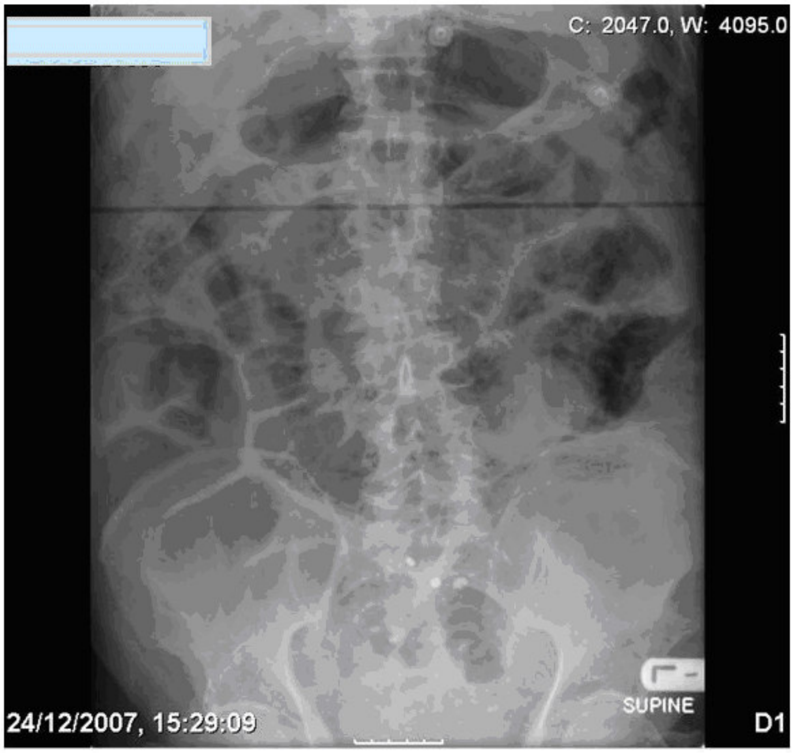

**FIGURE 2.** Abdominal X-ray showing dilated small and large bowels. The small bowel wall appears thickened.

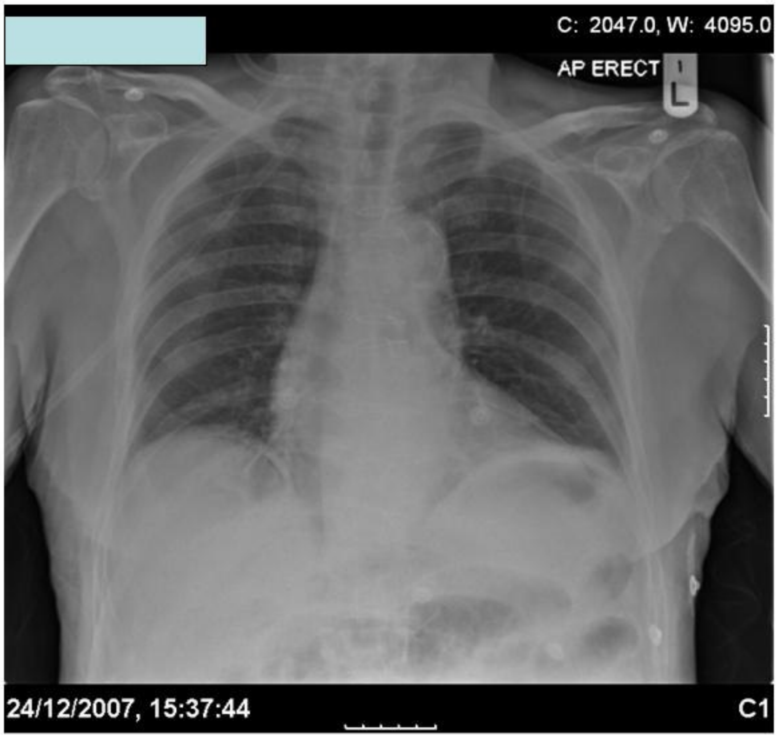

**FIGURE 3.** Chest X-ray showing air under the right hemidiaphragm.

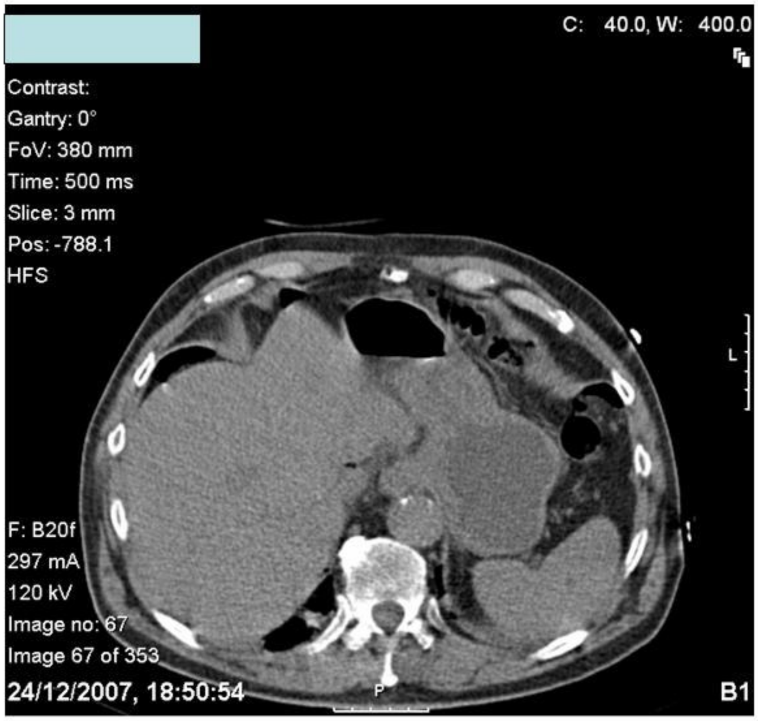

**FIGURE 4.** CT scan of the abdomen showing pneumoperitoneum.

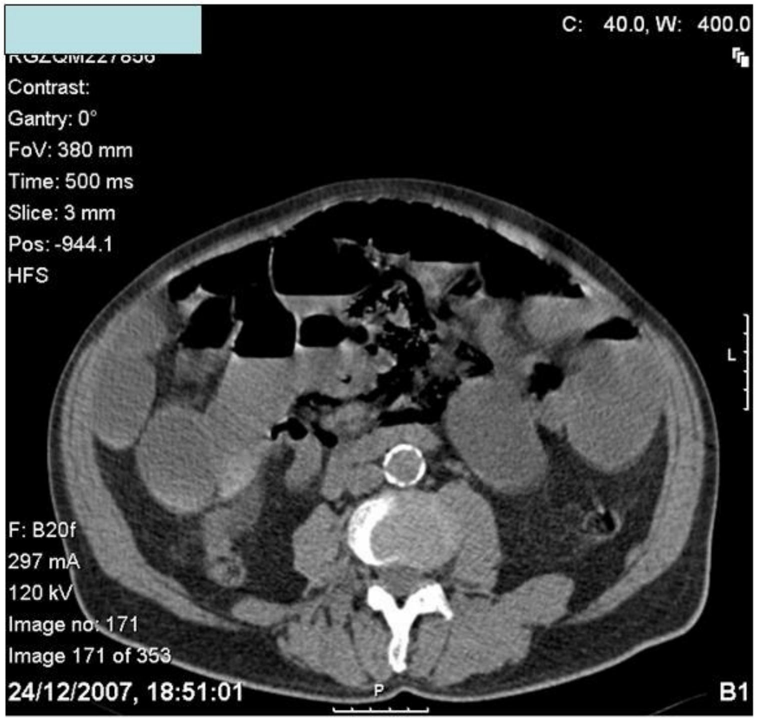

**FIGURE 5.** CT scan of the abdomen showing air fluid level in the small bowel.

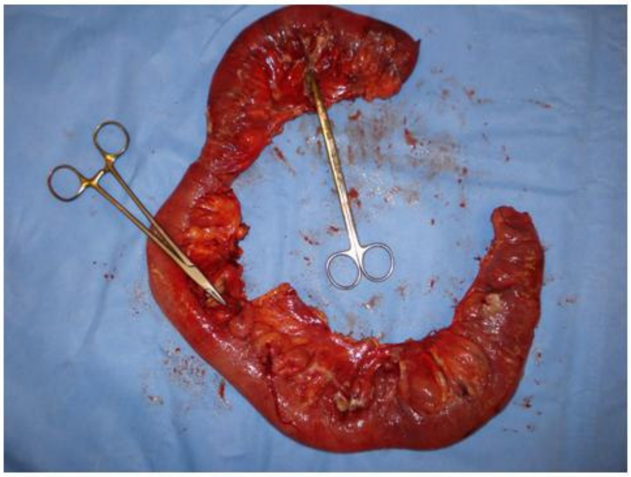

**FIGURE 6.** Resected jejunum containing multiple jejunal diverticula identified by the tip of the needle holder and rupture of one of them identified by the tip of the scissors.

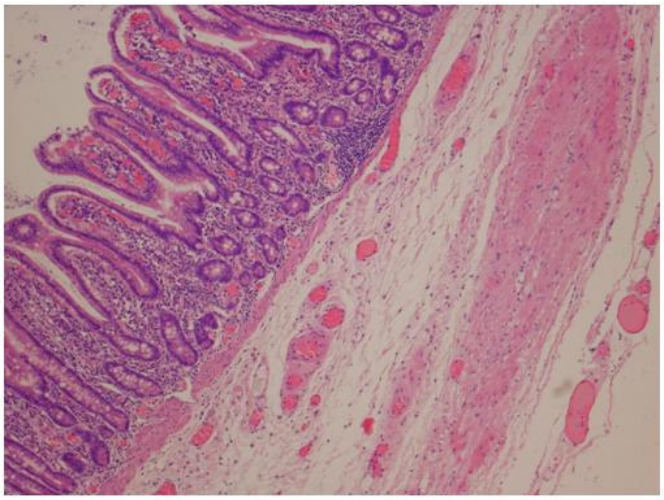

**FIGURE 7.** Low-power (×40) photomicrograph of a jejunal diverticulum showing all the layers of small bowel, namely mucosa, submucosa, muscular layer, and serosa. There is no sign of inflammation.

There may be attenuation of the muscularis propria[11], and the intestinal smooth muscle or myenteric plexus is often defective[8]. There may be an underlying motility disorder and intraluminar pressure may be high[12]. Angiodysplasia can cause bleeding in cases of jejunal diverticulosis[13]. Jejunal diverticulosis is reported in association with visceral neuropathy, which is common in connective tissue disease[14,15] such as SLE[16]. Small bowel diverticulosis can run in the same family, in particular with autoimmune disease[17].

Plain abdominal X-ray may not show jejunal diverticula. Enteroclysis or small bowel enema is the most reliable method of diagnosing small bowel diverticulosis[7,12]. Ultrasound examination may show multiple peri-intestinal hypoechoic structures which communicate with the intestinal lumen[18]. CT abdomen with oral contrast may also show typical diverticula. Since they are situated along the mesenteric border, it may be difficult to diagnose them without an oral contrast.

Jejunal diverticulosis is usually asymptomatic, and uncomplicated jejunal diverticulosis does not need any surgical intervention[19]. Enterolith can form inside the diverticulum and migration of the stone can cause intestinal obstruction[20,21,22,23]. It can present as gastrointestinal bleeding[24] and a capsular endoscopy[25], mesenteric angiography, red blood cell scan, or an intraoperative enteroscopy may help to find out the cause[26,27]. Other presentations include intra-abdominal abscess, perforation with diffuse peritonitis, small bowel volvulus[28,29], and jejunocolic fistula. Chronically, it can present as malabsorption syndrome.

According to the published literature, the prognosis of perforated jejunal diverticulum is related to the duration of symptoms before the operation, Patient's age, type of surgical procedure, and gender do not affect the outcome[30]. In a case series, out of 13 patients who underwent surgery for perforated jejunal diverticula, two died[31]. Our patient presented with only a 1-week history of abdominal pain prior to his operation. Even though he recovered from his operation, he eventually died from respiratory failure.

## SUMMARY

Whenever a patient with known diverticular disease of the colon presents with peritonitis, one should not assume that it is due to colonic diverticular perforation. Therefore, a systematic examination should be performed during the laparotomy. If a ruptured jejunal diverticulum is found, usually there will be a precipitating cause for the rupture, such as a band, stricture, or foreign body, and they should be treated accordingly.

## ACKNOWLEDGEMENT

The authors are grateful to Mr. N.J. Catlett, MSc, CSci, FIBMS, Senior Biomedical Scientist, Department of Cellular Pathology, Queen Mary's Hospital, Sidcup for helping us take the photograph of the histological sections.
